# Implementation and Impact of the WHO Integrated Care for Older People Program in Older Adults With Frailty in China

**DOI:** 10.1093/geroni/igaf122.1281

**Published:** 2025-12-31

**Authors:** Lin Kang

**Affiliations:** Peking Union Medical College Hospital, Beijing, Beijing, China (People’s Republic)

## Abstract

Fragmentation of services increases health and social care burden as people live longer with higher prevalence of diseases, frailty and dependency. Evidence from China and around the world indicates that development and implementation of person-centered integrated care are urgently needed to improve senior health and achieve healthy ageing. To test the feasibility and impact of World Health Organization’s (WHO) Integrated Care for Older People (ICOPE) approach in China, we designed a randomized controlled trial to evaluate its impact on health outcomes and health resource utilization. Community-dwelling older adults screened as at-risk of functional declines (pre-frailty or frailty) were randomized into intervention (n = 537) and control (n = 1611) groups between September 2020 and February 2021.A 6-month intervention program following WHO ICOPE care pathways implemented by integrated care managers compared to standard care. After 1 to 1 propensity score matching, participants in intervention and control groups (totally 938) had comparable baseline characteristics, demonstrated feasibility of implementing ICOPE with satisfaction by participants (97–99%) and providers (92–93%). All outcomes showed improvements after a 6-month intervention, with statistically significant least-squares mean differences (control-intervention) in vitality as measured by mini-Nutritional Assessment Short Form to measure vitality (−0.21, 95% CI, −0.40–0.02, worse in control group), mobility by Short Physical Performance Battery (−0.29, 95% CI, −0.44–0.14), and psychological health by 5-item Geriatric Depression Scale (0.09, 95% CI, 0.03–0.14). These results support the feasibility and impact of this intervention on health outcomes of frail older adults as well as its acceptance among key stakeholders in China.

